# Fragment-based identification of multi-target ligands by self-organizing map alignment

**DOI:** 10.1186/1758-2946-4-S1-P57

**Published:** 2012-05-01

**Authors:** Janosch Achenbach, Franca-Maria Klingler, Steffen Hahn, Svenja Steinbrink, Mirjam Schroeder, Frank Loehr, Volker Doetsch, Dieter Steinhilber, Ewgenij Proschak

**Affiliations:** 1 Institute of Pharmaceutical Chemistry, LiFF/OSF/ZAFES, Goethe University, Frankfurt am Main, Germany, Max-von-Laue Str.9, D-60438, Frankfurt/M, Germany

## 

In the recent years the prevalent paradigm in drug discovery of „one drug – one target – one disease“, following the assumption that highly selective ligands would avoid unwandted side effects caused by binding to seconday non-theratpeutic targets, got reconsidered. The results of post-genomic and network biology showed that proteins rarely act in isolated systems but rather as a part of a highly connected network [1]. It was further shown that the efficacy of several approved drugs is traced back to the fact that they act on multiple targets [2]. Therefore inhibiting a single target of such a network might not lead to the desired therapeutic effect. These findings lead to a shift towards polypharmacology [3] and the rational design of selective multi-target drugs, which have often improved efficacy [4]. But the design of multi target drugs is still a great challenge in regard of a sufficient activity on each target as well as an adequate pharmacokinetic profile [5]. Early design strategies tried to link the pharmacophors of known inhibitors, however these methods often lead to high molecular weight and low ligand efficiency.

We present a new approach based on self-organizing maps [3,6] (SOM) for the identification of multi-target fragments. We describe a workflow that initially identifies multi-target relevant substructures with a combination of maximum common substructure search and the alignment of multiple SOMs. Furthermore, these substructures are trained together with a fragment library on additional SOMs to find new multi-target fragments, validated by saturation transfer difference (STD)-NMR and biochemical assay systems. We used our approach for the identification of new dual-acting inhibitors of 5-Lipoxygenase (5-LO) and soluble Epoxide Hydrolase (sEH), both enzymes located in the arachidonic acid cascade and involved in inflammatory processes, pain and cadiovascular diseases.

## References

[B1] Jeong HM Barabási A-L Oltvai ZN Nature 2001 411 41 42 10.1038/35075138 11333967

[B2] Yildirim Ma Goh K-I Cusick ME Barabási A-L Vidal M Nature Biotechnology 2007 25 1119 1126 10.1038/nbt1338 17921997

[B3] Achenbach J Tiikkainen P Franke L Proschak E Future medicinal chemistry 2011 3 961 968 10.4155/fmc.11.62 21707399

[B4] Morphy R Rankovic Z J Med Chem 2005 48 6523 6543 10.1021/jm058225d 16220969

[B5] Morphy R Kay C Rankovic Z Drug discovery today 2004 9 641 651 10.1016/S1359-6446(04)03163-0 15279847

[B6] Kohonen T Biological Cybernetics 1982 43 59 69

